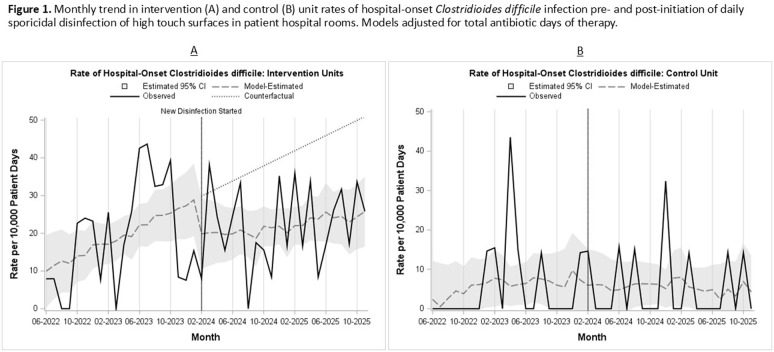# 125 A Qualitative Assessment of Antibiotic Stewardship Teams’ Efforts to Perform Prospective Audit-and-Feedback at Hospital Discharge

**DOI:** 10.1017/ash.2026.10538

**Published:** 2026-06-23

**Authors:** Cameron Griffin, Phillip Hahn, Jenny Chan Yuen, Elizabeth Phoung, Robyn Blacken, Lindsay Weir, Jennifer Ormsby, Ana Vaughan-Malloy, Thomas Sandora

**Affiliations:** 1 Boston Children’s Hospital; 2 Boston Children’s Hospital, Program for Patient Safety and Quality; 3 Boston Childrens Hospital

## Abstract

**Background:** In adult hospitals, daily disinfection using a sporicidal agent in patient rooms has resulted in sustained reduction in Clostridioides difficile infection (CDI) rates. No studies have evaluated the impact of this intervention in a pediatric setting. Objectives: We sought to evaluate the impact of daily sporicidal disinfection of high-touch surfaces in rooms of hospitalized pediatric oncology and stem cell transplant (SCT) patients on the incidence density of hospital-onset CDI, and to identify strategies to optimize reliability of disinfection by clinical staff. **Methods:** We introduced a quality improvement intervention in which nurses disinfected 6 high-touch surfaces in patient rooms with bleach wipes twice daily. PDSA cycles were used to improve reliability of disinfection. We used a controlled interrupted time-series analysis with a non-equivalent control group (pediatric ward housing solid organ transplant recipients in which no sporicidal disinfection was introduced) and also tracked a non-equivalent dependent variable (catheter-associated UTI rate) expected to be unaffected by the intervention. Autocorrelated segmented regression models were used to compare CDI rates in each period (pre: June 2022-January 2024, post: February 2024-November 2025), adjusting for total antibiotic days of therapy. Other potential confounders (including reliability of hand hygiene, personal protective equipment, and room cleaning) were also monitored. **Results:** Sociodemographic and clinical characteristics among encounters on intervention units were similar pre- and post-intervention. The overall CDI rate on the oncology and SCT units was 20.5 per 10,000 patient days with no difference pre- vs post-intervention (19.0 vs. 21.9, p=0.46). The average monthly reliability of sporicidal disinfection across the post-intervention period was 56%. The model-estimated monthly trend in CDI rate on the intervention units decreased from 1.00 (pre) to 0.32 (post) (p=0.24) and showed a greater but non-significant month-over-month decline in trend compared with the control unit (β=-0.28, p=0.74). Hand hygiene and other confounders were similar across periods. There was no change in control unit CDI trend pre- vs post-intervention (p=0.48) (Figure 1). The CAUTI rate did not change between periods on intervention or control units. **Conclusions:** Introduction of daily sporicidal disinfection of high-touch surfaces by nurses in the rooms of pediatric oncology and stem cell transplant patients was followed by a modest (but not statistically significant) decrease in monthly trend of hospital-onset CDI rate, despite only moderate reliability of disinfection. Our results suggest a potential benefit to including sporicidal disinfection as a routine component of CDI prevention in a pediatric hospital.

**Figure:**